# WO_3_ electrodes by spray pyrolysis for photoelectrochemical applications: impact of W precursor and Cl incorporation

**DOI:** 10.1039/d5ra07105d

**Published:** 2026-01-16

**Authors:** Mohammed M. Gomaa, Hana Krýsová, Mohamed H. Sayed, Tomáš Imrich, Mostafa Boshta, Michael Neumann-Spallart, Josef Krýsa

**Affiliations:** a Solid State Physics Department, National Research Centre 12622 Dokki Giza Egypt; b J. Heyrovský Institute of Physical Chemistry, Czech Academy of Sciences Dolejškova 2155/3 182 23 Prague 8 Czech Republic; c Molecular and Fluorescence Spectroscopy Lab., Central Laboratories Network, National Research Centre 12622 Dokki Giza Egypt; d Department of Inorganic Technology, University of Chemistry and Technology Prague Technická 5 166 28 Prague 6 Czech Republic Josef.Krysa@vscht.cz

## Abstract

This study explores the influence of various precursors including ammonium metatungstate (AMT) and peroxotungstic acid (PTA) in water, and tungsten hexachloride (WCl_6_ in MeOH or EtOH), as well as the role of ammonium chloride incorporation on the structural, morphological, and photoelectrochemical characteristics of WO_3_ layers synthesized by spray pyrolysis. X-ray diffraction (XRD) analysis revealed that films annealed at 550 °C crystallized in the monoclinic phase of WO_3_ with a polycrystalline structure without amorphous parts. Different morphological features of the samples were identified by scanning electron microscopy (SEM): dense grains for films formed using PTA, aggregated grains for films synthesized from AMT, smooth and uniform surfaces for films based on WCl_6_, and porous architectures resulting from NH_4_Cl incorporation. Photoelectrochemical measurements under UV and simulated solar illumination demonstrated that AMT/NH_4_Cl – derived WO_3_ films significantly enhanced the initial photocurrent density, reaching values of up to ∼3 mA cm^−2^ under UV light. Topological energy dispersive spectroscopy (EDS) revealed the existence of Cl rich areas responsible for this effect. With prolonged exposition to light and bias, Cl in these areas was oxidatively exhausted and average current densities as in samples obtained with other precursors were obtained. These findings highlight the critical role of precursor selection and doping in determining the photoelectrochemical performance of spray-deposited WO_3_ photoanodes.

## Abstract 2

Long-term chronoamperometry under irradiation (AM 1.5 (1 sun)) of WO_3_ films at 1.4 V *vs.* Ag/AgCl. The decrease of photocurrent is explained by the oxidative exhaustion of Cl^−^ rich areas present in WO_3_ films as proved by EDS analysis.

## Graphical abstract

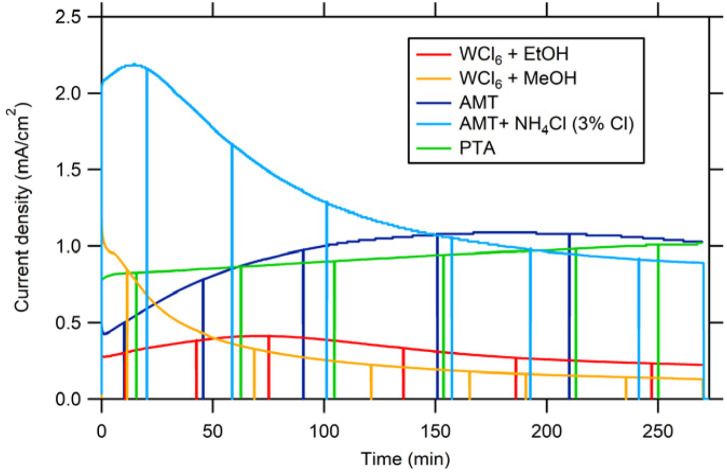

## 1. Introduction

Photocatalysis and photoelectrochemical (PEC) techniques are the subject of significant studies due to the growing need for clean environmental solutions and sustainable energy sources. Applications include CO_2_ reduction, organic pollutant degradation, and photoassisted water electrolysis for hydrogen generation. Semiconductor based systems can use solar energy for generating electricity or valuable chemicals.^1–6^ The development of effective, reliable, and eco-friendly semiconductor materials that can absorb visible light and produce charge carriers for redox reactions is essential for these processes.^7–9^ Such materials require an appropriate bandgap to effectively capture sunlight, photostability, good carrier mobility, and non-toxicity.^10,11^ Over the last few decades, many metal oxides have been investigated as photoelectrode materials for PEC applications. n-type semiconductors such as TiO_2_, ZnO, and Fe_2_O_3_ have gained popularity due to their low cost, abundance, and chemical stability.^12,13^ Considering these advantages, each of these materials has significant drawbacks that limit overall PEC efficiency. TiO_2_ and ZnO have wide band gaps, restricting their ability to capture visible light. Fe_2_O_3_ has a valence band energy that excludes oxidation of impurities requiring a high potential of the valence band.^14^ These limitations encouraged the development of semiconductors with improved optical and electrical properties.^15,16^ In this context, WO_3_ has distinguished itself as a promising semiconductor. Its band gap of 2.7 eV (ref. 17) enables absorption of part of the visible light, making it suited for various applications.^18–20^ Beyond its optical properties, WO_3_ exhibits efficient charge carrier transport, good chemical stability in acidic environments, and strong resistance to photocorrosion.^19,21,22^ These combined advantages have led to growing interest in WO_3_ for a range of applications, including photoassisted water electrolysis, photocatalytic degradation of organic pollutants, and gas sensing.^18,19,23–25^

An effective strategy for further improving the PEC performance of WO_3_ is the modification of its physical properties by additives influencing growth and structure of layers. Electronic properties can be influenced by doping with metal or non-metal atoms.^5,6,15,25^ Doping by inducing oxygen vacancies or by foreign atoms has the purpose of increasing the conductivity of the material if easily ionizable donor states (in the case of an n-type material) can be formed, thereby shifting the Fermi level and influencing the space charge layer width.^18,20,26–32^

Previous research has shown that doping WO_3_ with metal and non-metal elements improved photoelectrochemical performance.^30,32^ A deeper understanding of Cl doping and the resulting structural and electronic modifications in WO_3_ could provide valuable insights for enhancing its PEC performance. Chlorine doping presents a challenge to change the electronic and structural properties of WO_3_. Chlorine, as a monovalent anion, can substitute for oxygen or be interstitially incorporated into the lattice, leading to the formation of oxygen vacancies and modified defect chemistry.^33–35^ However, the challenge lies in achieving uniform and controlled doping, which strongly depends on the synthesis method used. In addition, the method of material preparation plays a critical role in determining the morphology, crystallinity, dopant distribution, and ultimately the PEC performance of WO_3_. Various synthesis techniques such as sol–gel processing,^36^ hydrothermal synthesis,^28,37^ electrodeposition,^38^ chemical vapor deposition,^39^ doctor blading,^40^ brush painting,^40^ spin coating,^41^ drop-casting,^42^ spray pyrolysis^43^ and aerosol pyrolysis^44,45^ have been employed to fabricate WO_3_ films. Each technique offers unique advantages and limitations in terms of scalability and control over film characteristics. Among these, spray pyrolysis (SP) stands out as a particularly attractive method for preparing doped WO_3_ thin films. SP is a simple, low-cost, and scalable technique that involves spraying a precursor solution onto a heated substrate, where it undergoes thermal decomposition to form a film.^18,25,28,31^ This method allows precise control over film thickness, morphology, and composition by tuning parameters such as solution concentration, spray rate, substrate temperature, and carrier gas flow. Furthermore, SP is well-suited for incorporating dopants by simply modifying the precursor solution, making it ideal for doping studies. Various precursors in organic, *e.g.* WCl_6_ in dimethylformamide^46^ and aqueous *e.g.* (ref. 45 and 47) solvents and additives (*e.g.* In (ref. 46)) have been used. In addition to synthesis parameters, the selection of the precursor plays a decisive role in determining the final structural, morphological, and photoelectrochemical properties of WO_3_ films.

In this work, we intended to fabricate WO_3_ thin films on fluorine-doped tin oxide (FTO) substrates using SP, employing different starting materials, such as ammonium metatungstate (AMT), peroxotungstic acid (PTA), and WCl_6_ and to evaluate their photoelectrochemical performance. In particular, the study focuses on the effect of chlorine incorporation using ammonium chloride as a Cl source on the structural, morphological, and photoelectrochemical properties of WO_3_ films. Special attention was given to the long term performance of the WO_3_ films under irradiation and electrical bias. The findings were expected to be helpful with the rational design and optimization of improved photoelectrode materials for environmental applications such as the remediation of organic impurities in water.

## 2. Experimental

### 2.1. Materials and electrodes

All chemicals used in this study were of analytical grade and purchased from Sigma-Aldrich without any further purification. FTO coated glass with a sheet resistance of 7 Ω sq^−1^, was purchased from Sigma-Aldrich and used as substrate for film deposition. Prior to deposition, 5 cm × 1 cm sized substrates were ultrasonically cleaned in acetone, ethanol and water with each step lasting for 10 minutes, to ensure the removal of surface contaminants and achieve good film adhesion.

WO_3_ thin films were deposited *via* SP under ambient air conditions. The SP apparatus has been described previously.^37^ It employed an automatic spray gun from FUSO SEIKI Co., Ltd; Japan. The distance between the nozzle and the substrate was 30 cm. The flow rate of the precursor solution was 0.3 ml min^−1^. Deposition was done by spraying the solution containing the precursor solution onto preheated substrates. Different tungsten based precursor solutions were prepared: AMT and PTA in water and WCl_6_ in MeOH or EtOH. In some experiments, NH_4_Cl was added in varying molar ratios to the AMT-based precursor solution. A summary of the precursor types, solvents, and deposition conditions used for WO_3_ film fabrication is provided in Table 1.

**Table 1:** Deposition parameters of WO_3_ films by SP

Precursor	Solvent	Nominal deposition temp. (°C)
0.1 M PTA	Water	450
0.1 M AMT	Water	450
0.1 M AMT + 0.003 M and 0.006 M NH_4_Cl	Water	450
0.1 M WCl_6_	Ethanol	350
0.1 M WCl_6_	Methanol	350

Film thickness was controlled by adjusting the deposition time and was quantitatively measured using a Dektak XT profilometer (Bruker) equipped with Vision 64 software, utilizing a 2 µm stylus tip with a 4 mg stylus force. The structural properties of the prepared films were analyzed using XRD, conducted on a PANalytical X'Pert PRO diffractometer equipped with a Cu K_α_ radiation source (*λ* = 1.5406 Å) and a 1D X'Celerator detector. The measurements were carried out at an accelerating voltage of 40 kV, a current of 30 mA, and a step size of 0.0390° in 2*θ*. Surface morphology features of the deposited films were examined by field emission scanning electron microscopy (FE-SEM) using a Hitachi FE-SEM 4800 system (Japan). Compositional analysis was done with a Flexsem (Hitachi) machine using a silicon drift detector (SDD) for EDS analysis.

### 2.2. Photoelectrochemical characterization

The photoelectrochemical cell was equipped with a fused silica optical window and was controlled by a Zahner workstation. Films were illuminated from the front side (“EE” – electrode/electrolyte interface) by a UV LED diode (LS365-2), center wavelength 369 nm, with an irradiance of 100 W m^−2^. For polarograms, irradiation was applied with 5 s dark/light intervals. Photoelectrochemical measurements were performed in 0.1 M HClO_4_.

## 3. Results and discussion

### 3.1. Film growth and thickness

**Fig. 1:**
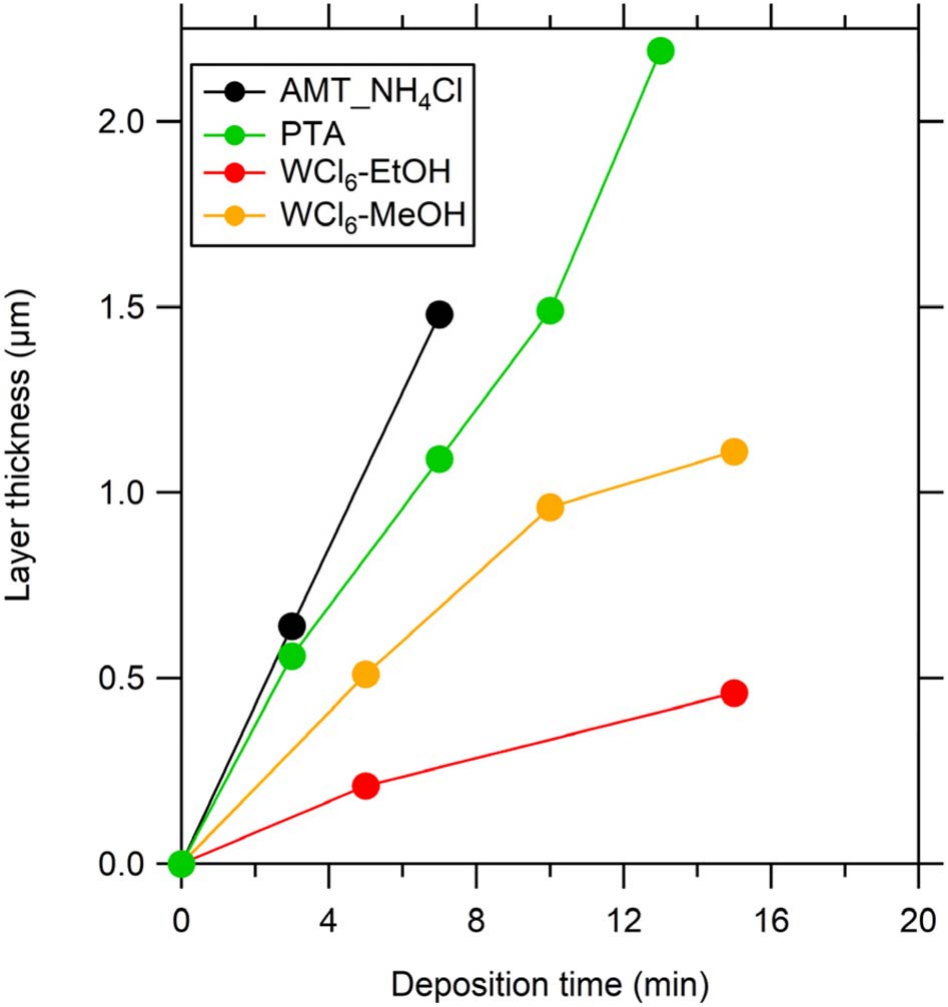
Dependence of WO_3_ thin film thickness on the deposition time for various precursor solutions: (i) AMT-NH_4_Cl precursor, (ii) PTA precursor and (iii) WCl_6_ precursor (all as-deposited films were annealed in air at 550 °C/4 h).

Films were prepared by spray pyrolysis of various precursor solutions. The susceptor temperature set-point was 450 °C. Total amounts of 0.9 to 4.5 ml were sprayed. Following deposition, all WO_3_ thin films were annealed in air at 550 °C for 4 hours to improve crystallinity and mechanical stability. Fig. 1 illustrates the relationship between film thickness and deposition time for WO_3_ thin films using different tungsten-based precursors. Different growth rates were observed for films derived from AMT, WCl_6_, and PTA. This can be attributed to differences in the thermal decomposition kinetics of each precursor on the heated substrate surface. Specifically, factors such as the volatility of the solvent and conversion efficiency of the precursor to WO_3_ significantly influence the nucleation and growth dynamics, resulting in distinct film growth rates and final thicknesses.^3^ In conclusion, the chemistry of the precursor had a significant impact on the kinetics of deposition and the final film morphology in SP.

### 3.2. Structural analysis

**Fig. 2:**
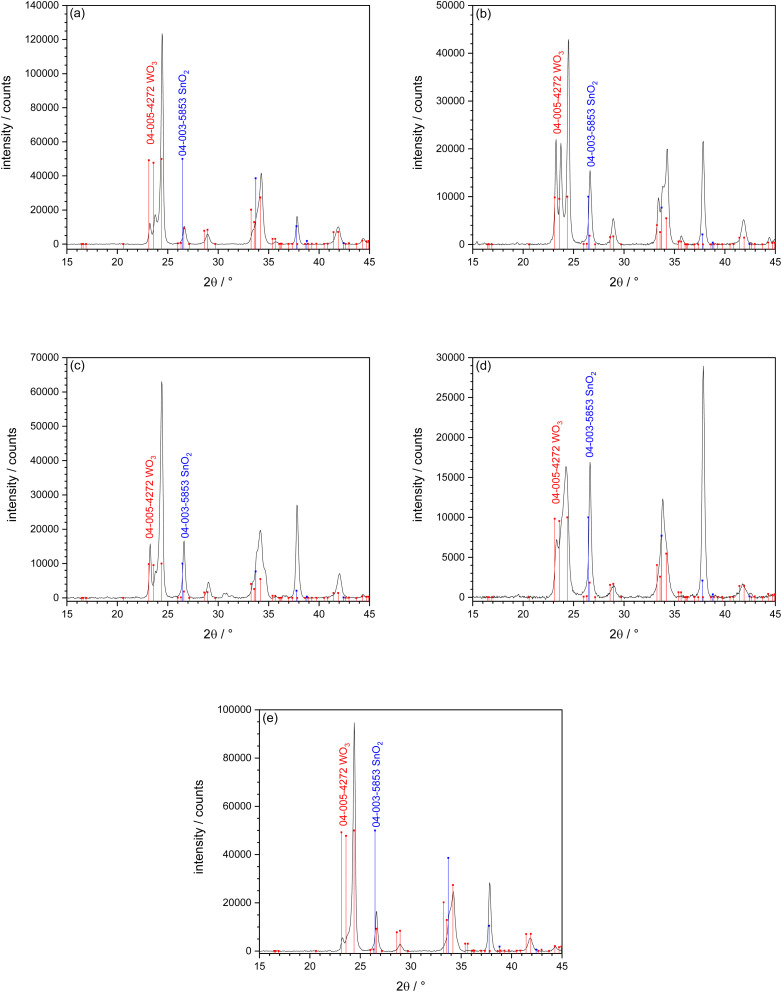
XRD of WO_3_ films on FTO/glass obtained by SP: (a) precursor AMT, thickness 0.71 µm, (b) precursor AMT + 3 mol% NH_4_Cl, thickness 0.64 µm, (c) precursor PTA, thickness 0.54 µm, (d) precursor WCl_6_ in MeOH, thickness 0.51 µm, (e) precursor WCl_6_ in EtOH, thickness 0.43 µm. All samples were annealed at 550 °C for 4 h in air. XRD reference lines 04-003-5853 cassiterite (SnO_2_, substrate peaks) and 04-005-4272 tungsten trioxide (WO_3_).^48^

Fig. 2 shows the XRD patterns of WO_3_ films deposited with various precursor solutions, followed by thermal annealing at 550 °C for 4 hours in air. All sprayed films showed diffraction peaks corresponding to the monoclinic crystal structure of WO_3_, fitting the reference data (JCPDS card no. 04-005-4272). The obtained diffraction patterns were in good agreement with previously reported studies, confirming the successful formation of polycrystalline monoclinic WO_3_.^3,47^ These findings showed that the monoclinic phase can be reliably produced using different tungsten based precursors. Among the various films, the one prepared using the AMT precursor showed the highest diffraction peak intensities. The sharp peaks in this sample indicate good crystal quality and preferred orientation. In contrast, WO_3_ thin films synthesized using PTA and WCl_6_ precursors exhibited lower diffraction peak intensities. This suggests differences in the thermal decomposition of the precursors or variations in film growth kinetics during the pyrolysis process. The WO_3_ film deposited by AMT containing NH_4_Cl in the precursor solution had low peak intensities compared to WO_3_ deposited from a pure AMT precursor solution and had different preferred growth orientation. It can be seen that the NH_4_Cl doping influenced the growth direction and the preferred orientation of the WO_3_ film. Further analysis was carried out on WO_3_ films deposited using WCl_6_ dissolved in either MeOH or EtOH. The film sprayed using the MeOH based precursor was similar to the one deposited with EtOH as solvent.

### 3.3. Morphological analysis

**Fig. 3:**
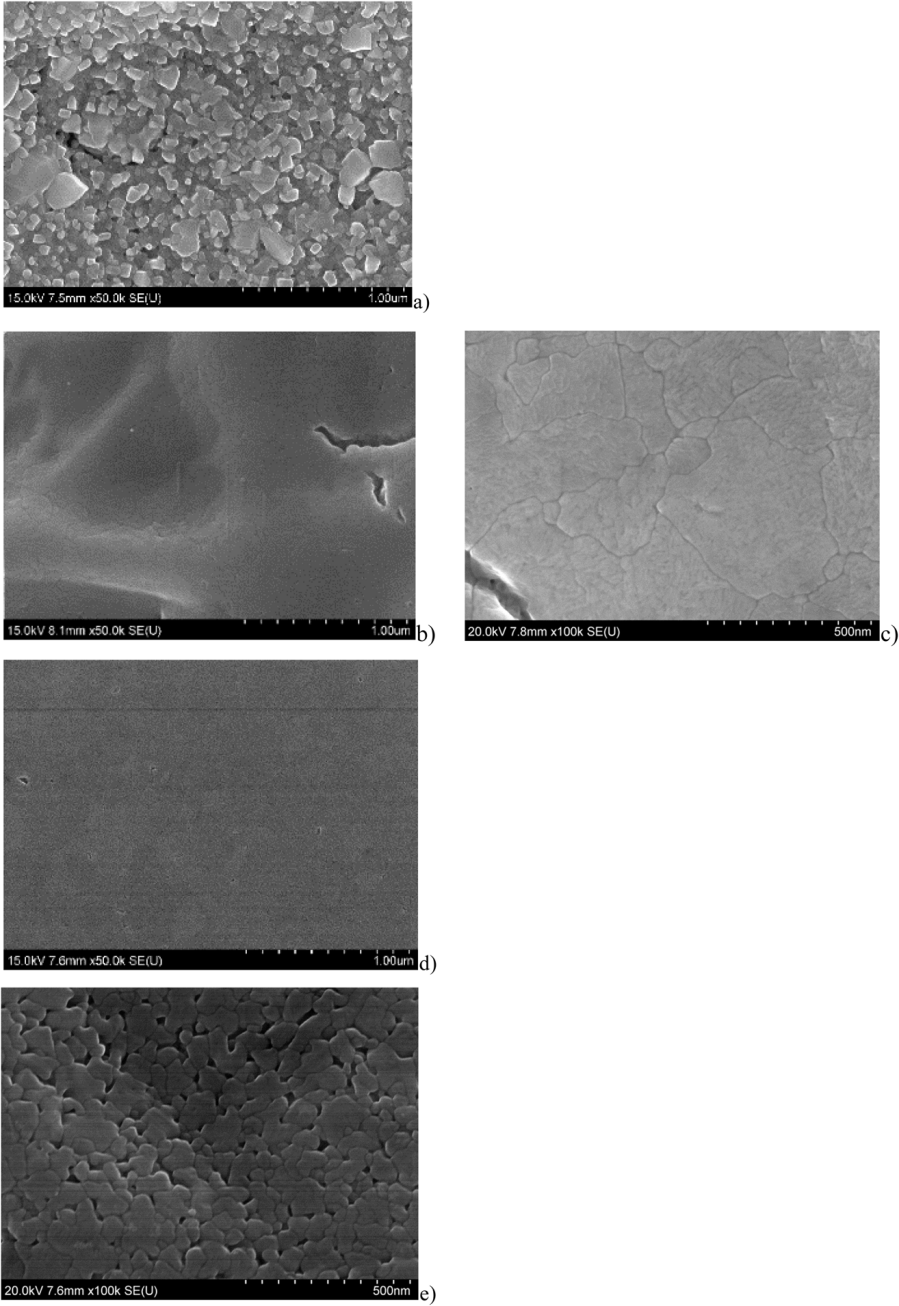
Top view SEM morphology of WO_3_ films prepared by spray deposition with (a) PTA precursor, 0.56 µm, (b) and (c) AMT precursor, 0.58 µm, (d) WCl_6_ in EtOH precursor, 0.46 µm, and (e) AMT + NH_4_Cl (3 mol% Cl in the precursor solution).

Top view SEM images of WO_3_ films prepared by spraying of different precursor solutions are shown in Fig. 3. The SEM micrograph (a) of the WO_3_ film prepared with the PTA precursor was composed of a dense agglomeration of 100 to 200 nm sized particles.

The SEM image of the WO_3_ thin film prepared using AMT as a precursor (b, c) had a dense appearance. The porosity was noticeably reduced compared to the film prepared from the PTA precursor. The WO_3_ film prepared using WCl_6_ in EtOH as a precursor (d) showed a smooth, dense, and homogeneous morphology compared to the other precursors. The SEM micrograph of the film prepared AMT doped with NH_4_Cl (e) revealed a smooth surface and well-connected grains with some porosity. The addition of NH_4_Cl seems to have resulted in a smaller primary grain size compared to the film prepared with pure AMT. NH_4_Cl likely acted as a modifying agent during the film formation, influencing the decomposition of the precursor and the subsequent growth and sintering of the WO_3_ nanoparticles.

The elemental composition of chloride-doped WO_3_ thin films was studied using energy dispersive X-ray spectroscopy (EDS). For instance, a 0.25 µm thick film deposited from AMD showed the following distribution: W 20.8 at%, O 72.8 at%, Sn 6.4 at%. The ratio of the atomic fractions of W, Sn and O show the stoichiometric occurrence of WO_3_ and the underlying substrate, SnO_2_.

### 3.4. Photoelectrochemical characterization

**Fig. 4:**
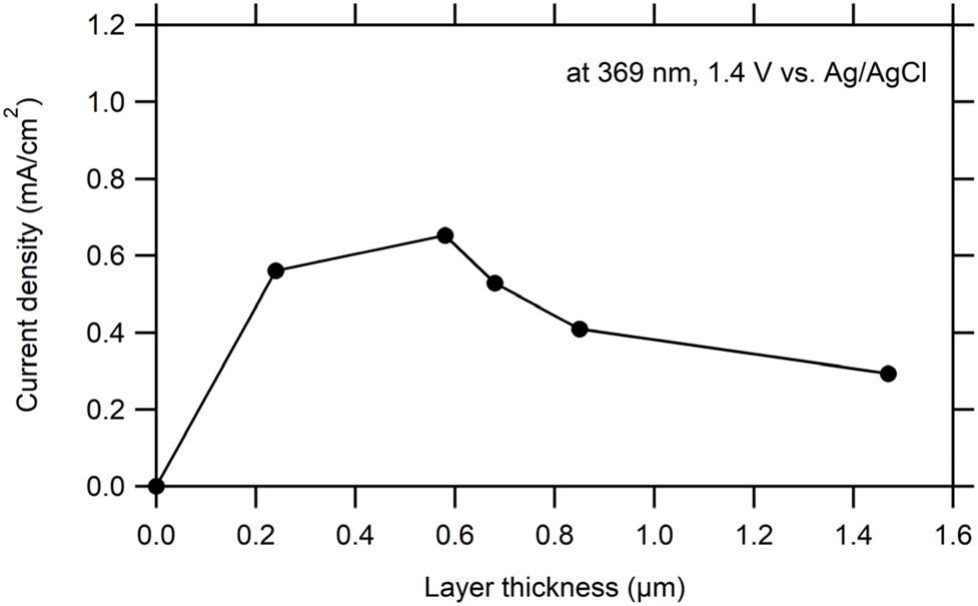
Photocurrent densities (at 1.4 V *vs.* Ag/AgCl) of WO_3_ samples prepared from an AMT precursor as a function of layer thickness. Electrolyte 0.1 M HClO_4_. UV LED (369 nm) as light source, irradiance 100 W m^−2^.

Fig. 4 shows the dependence of photocurrent densities of WO_3_ films obtained from the AMT precursor on layer thickness. It can be seen that increasing layer thickness up to 0.6 µm resulted in an increase of photocurrent densities (maximum 0.65 mA cm^−2^). With increasing layer thickness there was a decrease (to 0.3 mA cm^−2^).

**Fig. 5:**
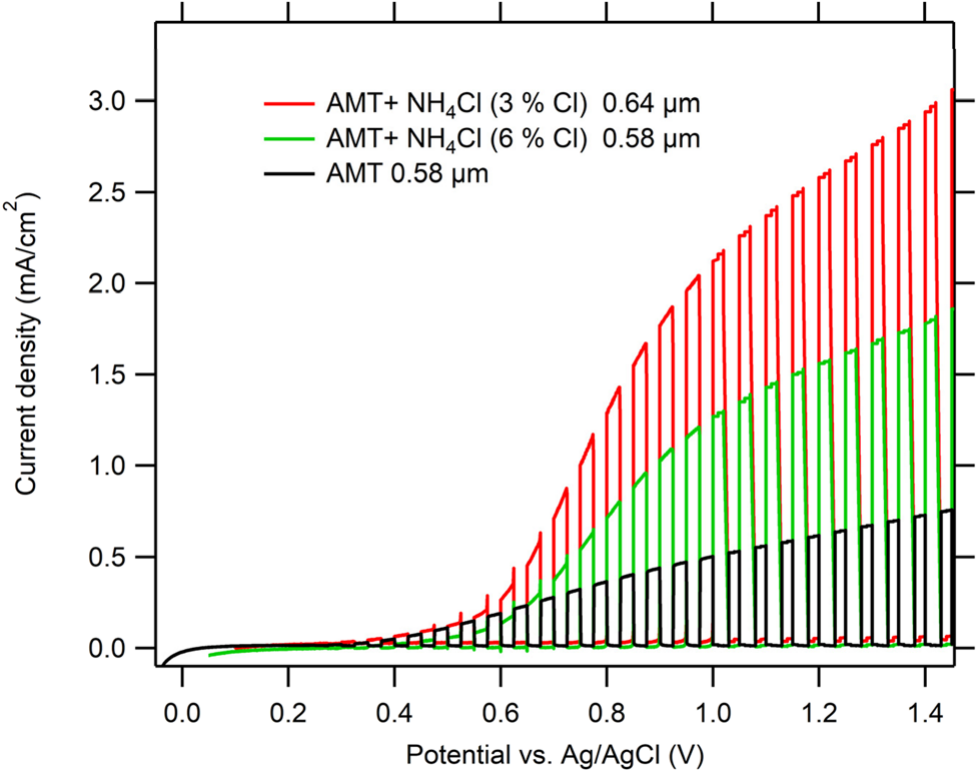
Chopped-light polarization curves of WO_3_ films spray deposited from AMT precursor with various concentrations of Cl in the precursor solutions (3% and 6%) and annealed at 550 °C/4 h. Electrolyte 0.1 M HClO_4_, scan rate 5 mV s^−1^. UV LED (369 nm) as light source, irradiance 100 W m^−2^, dark/light interval 5 s.

Comparison of chopped-light polarization curves of WO_3_ films (similar layer thickness) prepared from AMT precursor and modified AMT precursor is shown in Fig. 5. The photocurrent density of a WO_3_ film prepared from a modified AMT precursor (3% NH_4_Cl) was 4 times higher than that of a WO_3_ film prepared from AMT alone. Photocurrent densities of a WO_3_ film prepared from a precursor solution with 6% NH_4_Cl were only 2.4 times higher than of the pure AMT samples, showing the disturbance of the crystalline structure with such a large amount of a foreign impurity.

#### 3.4.1. Comparison of various precursors

**Fig. 6:**
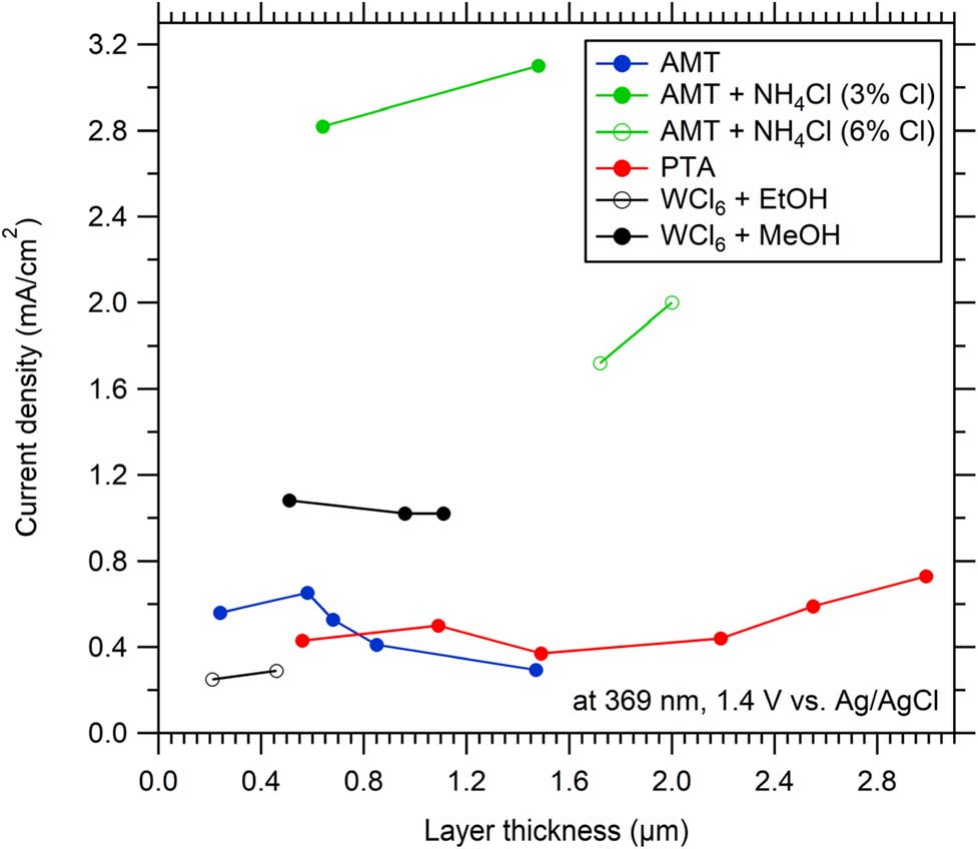
Initial photocurrent densities (at 1.4 V *vs.* Ag/AgCl) of WO_3_ samples prepared from various precursors as a function of layer thickness. Electrolyte 0.1 M HClO_4_. UV LED (369 nm) as light source, irradiance 100 W m^−2^.

Table S1 (in SI) and Fig. 6 summarize photocurrent densities of WO_3_ films of all deposited samples from all precursors at 1.4 V *vs.* Ag/AgCl under monochromatic illumination at 369 nm. For WO_3_ obtained from SP of WCl_6_ in EtOH, there was a small increase of the photocurrent density from 0.25 to 0.29 mA cm^−2^ with increasing layer thickness from 0.21 to 0.46 µm. Photocurrent densities of WO_3_ samples obtained from WCl_6_ in MeOH were much higher than those of samples prepared with EtOH. The WO_3_ (WCl_6_ in MeOH) sample deposited for 5 minutes had a similar thickness as WO_3_ (WCl_6_ in EtOH) deposited for 15 minutes (0.51 µm and 0.46 µm) but its (WCl_6_ in MeOH) photocurrent density was almost 4 times higher than that for WO_3_ (WCl_6_ + EtOH) (1.08 *vs.* 0.29 mA cm^−2^). Comparing pure precursors, the highest photocurrents were observed for the WCl_6_ in MeOH precursor.

Elevated photocurrent densities (2–3 mA cm^−2^) were only observed for WO_3_ films prepared from the NH_4_Cl modified AMT precursor. Such photocurrent densities slightly increase (about 10%) with layer thickness (for both NH_4_Cl concentrations). The highest observed photocurrent density of WO_3_ films (precursor AMT + 3% NH_4_Cl) was ∼3 mA cm^−2^.

The photocurrent density of WO_3_ films as a function of layer thickness for all used precursors showed that there was no significant influence of layer thickness above 0.5 µm on the photocurrent density when a light source with 369 nm center wavelength was used. This is because there is complete light absorption under these conditions. *E.g.* the penetration depth in typical WO_3_ films at 369 nm was around 0.5 µm.^41^

#### 3.4.2. Amperometry under simulated AM1.5G irradiation

**Fig. 7:**
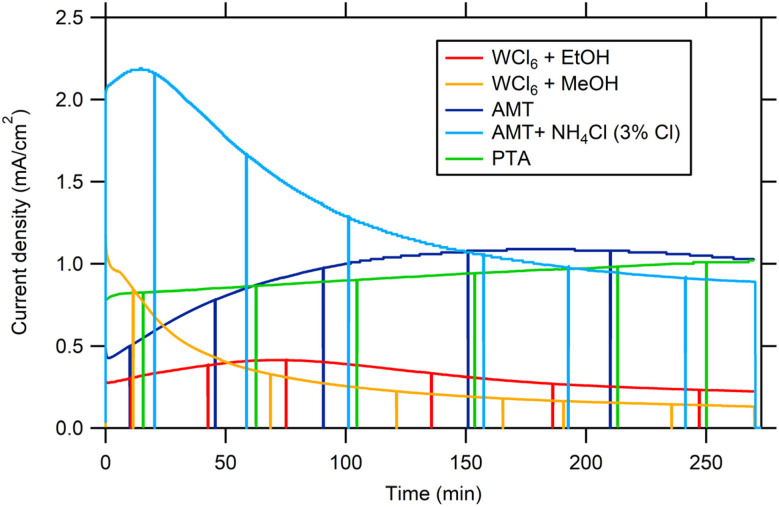
Long-term chronoamperometry of WO_3_ films (layer thickness between 0.5 and 0.6 µm) at 1.4 V *vs.* Ag/AgCl. Solar simulator, irradiance AM1.5 (1 sun), electrolyte 0.1 M HClO_4_.

Fig. 7 shows the long-term amperometry of 0.5–0.6 µm thick WO_3_ films made from different precursors at 1.4 V *vs.* Ag/AgCl under illumination with a solar simulator (AM1.5 G, 100 mW cm^−2^). The initial photocurrents (between 0.5 and 2 mA cm^−2^) are comparable to values reported in the literature under AM1.5 irradiation, *viz.* Fatty *et al.*^49^ and references therein and to photocurrents of WO_3_ films fabricated by aerosol pyrolysis (1.4 mA cm^−2^ (ref. 45)), hydrothermal treatment (2 mA cm^−2^ (ref. 3)) and spray pyrolysis (1.8 mA cm^−2^ (ref. 47)).

The photocurrent density of WO_3_ films prepared with the PTA precursor moderately increased after 4 hours (from 0.8 to 1 mA cm^−2^). In the case of the AMT precursor, the photocurrent density gradually increased with polarization time from 0.5 to 1 mA cm^−2^ after 4 hours.

When 3 or 6% (Cl : W) of NH_4_Cl was added to the AMT precursor solution, the initial photocurrent density of WO_3_ films was 2.1 mA cm^−2^ (about 3 times higher than for the pure AMT precursor), but upon further polarization and illumination it decreased by about 50% after 4 hours, reaching 0.9 mA cm^−2^. A possible explanation could be the photooxidation of chloride incorporated in the film due to incomplete decomposition of the precursor solution and leaching to the electrolyte. The enhancement of photocurrents due to the presence of Cl^−^ in the electrolyte was also reported by Koo *et al.*^50^ In order to check the hypothesis of Cl incorporation, topological (SEM) and compositional analysis by EDS was resumed.

**Fig. 8:**
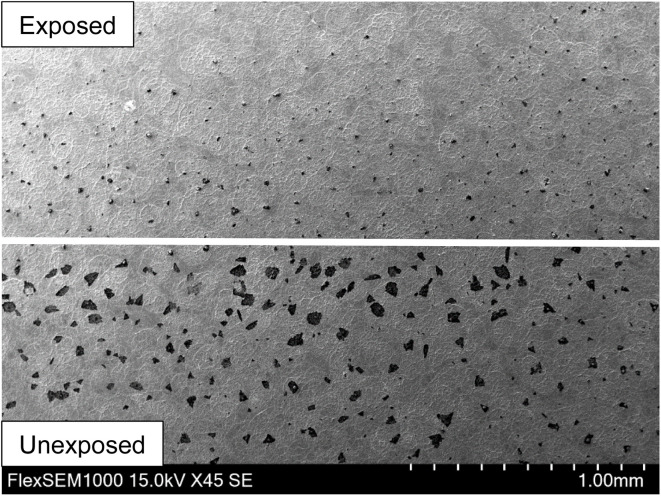
Influence of photoelectrochemical treatment of a WO_3_ electrode. Lower part … unexposed, upper part … after illumination under bias, passing 21 C cm^−2^.

**Fig. 9:**
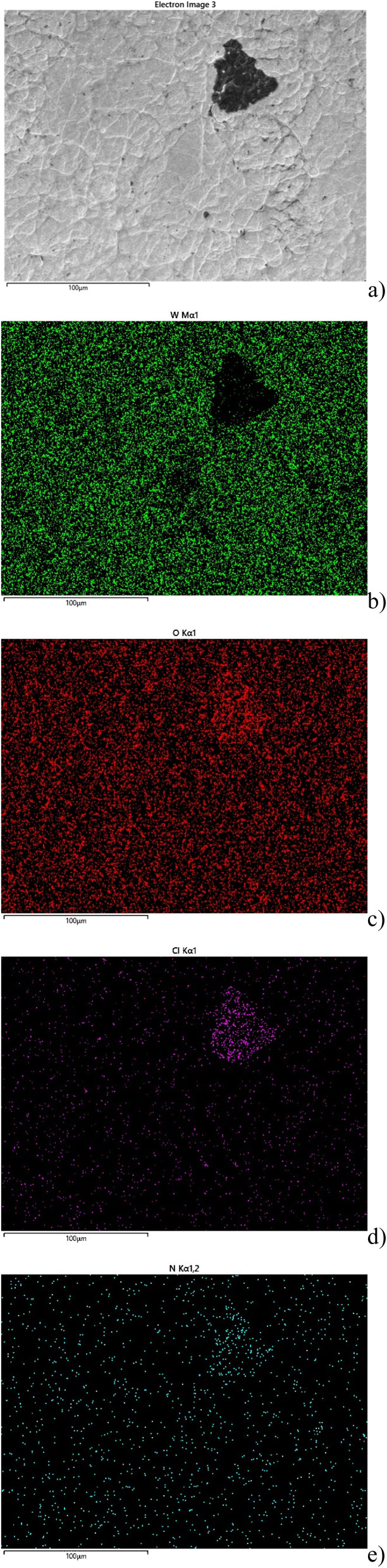
Mapping of elements on the surface of the films using false colours: (a) top view SEM, (b) W, (c) O, (d) Cl, (e) N.

**Fig. 10:**
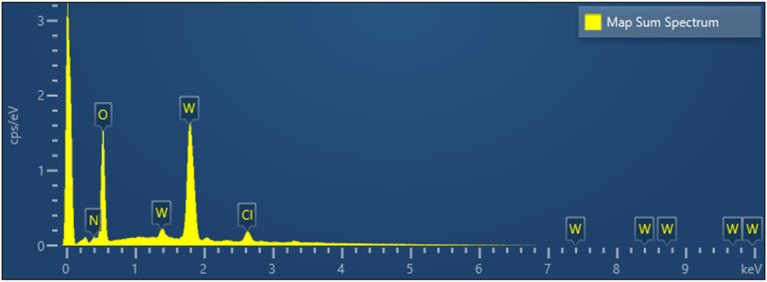
EDS spectrum of elements present on the surface of the spot.

Fig. 8 shows the presence of irregularly shaped dark spots in the unexposed (lower) part of a sample. After illumination under bias, these spots disappeared (upper part). In order to reveal their chemical composition, EDS analysis was carried out. Topological information is presented in Fig. 9 using false colours. Spots with a very high concentration of Cl (d) accompanied by N (e) were found on the surface. A quantitative compositional analysis of the spots was made (Fig. 10).

Fig. 10 shows the spectrum of elements and Table 2 corresponding content of elements on the spot shown in Fig. 9a. The ratio of Cl and N is close to 1. This may be attributed to accumulation of NH_4_Cl which was used in the precursor solution and was incompletely decomposed in the spray and the following annealing process. The absence of the signal of W (Fig. 9b) means that WO_3_ was covered in such areas.

**Table 2:** Content of elements present on the surface of the spot

Element	Line type	at%
W	M series	18.7
O	K series	67.2
Cl	K series	6.5
N	K series	7.6

As Cl^−^ was consumed, the photocurrent density (Fig. 7, blue curve) decreased accompanied by the disappearance of the black spots (Fig. 8), proving the hypothesis. A similar decrease in photocurrent density during polarization under light was observed for WO_3_ films fabricated from WCl_6_ in methanol (Fig. 7, yellow curve). Topological EDS analysis showed the presence of Cl rich areas as in the case of the AMT precursor modified with NH_4_Cl (Fig. 8, lower part) but to much smaller extent. Interestingly, WO_3_ films fabricated from WCl_6_ in ethanol (Fig. 7, red curve) do not show any decrease in photocurrent density during polarization under light and topological EDS analysis showed that the occurrence of Cl rich areas was almost negligible. In sum, all photocurrent densities converged to the same value after 270 min. of irradiation, namely around 1 mA cm^−2^, except for the WCl_6_ precursor. The lower photocurrents of films made with the WCl_6_ precursors cannot be explained presently and will be the subject of further research.

**Fig. 11:**
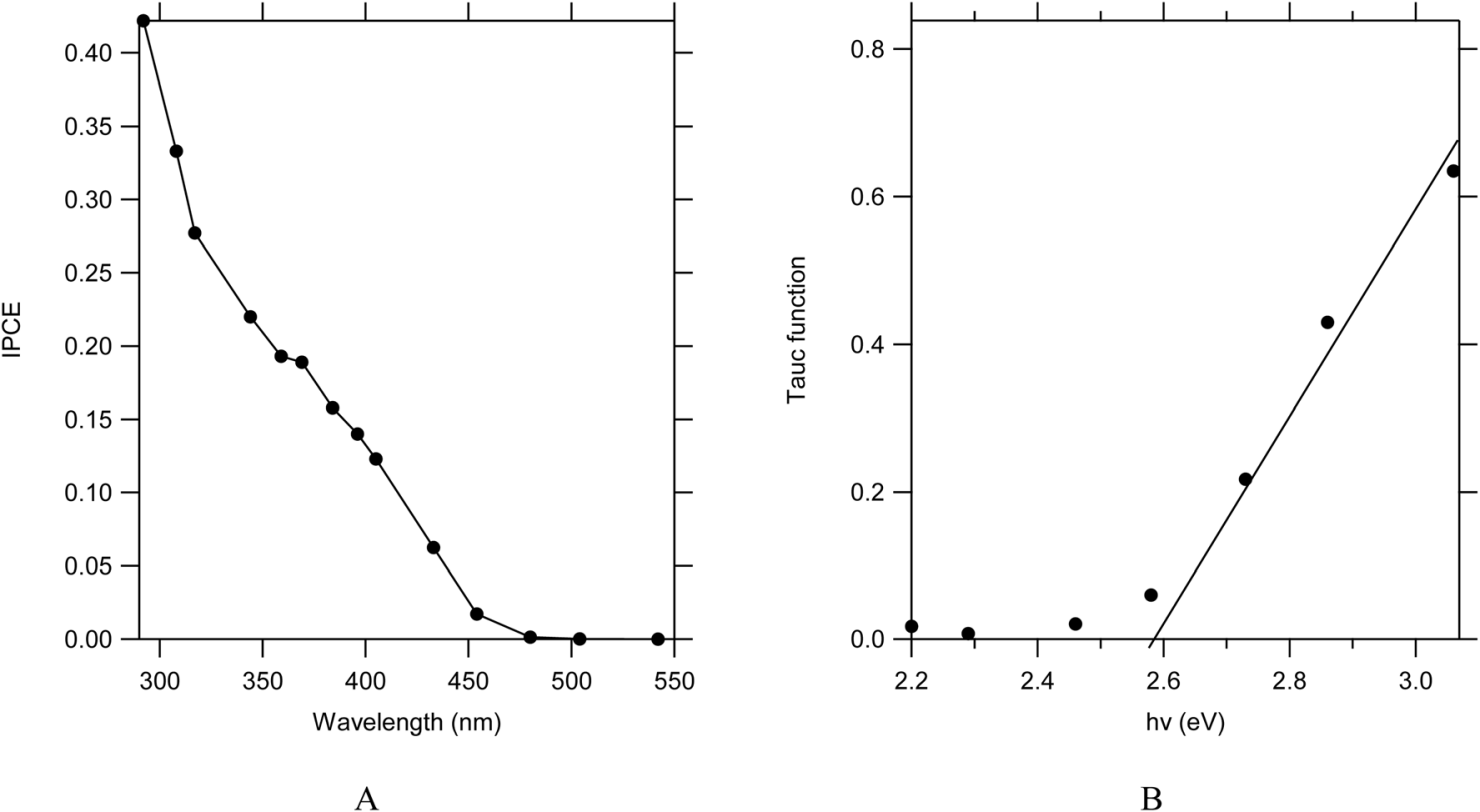
(A) IPCE as a function of wavelength for a 0.58 µm thick WO_3_ (AMT precursor) film at 1.3 V *vs.* Ag/AgCl in 0.1 M HClO_4_, (B) Tauc plot calculated as (ln(1/(1 − IPCE)) × *hν*)^1/2^ for an indirect transition.

WO_3_ films prepared from the AMT precursor were further investigated and Incident Photon-to-Current Efficiency (IPCE) as a function of wavelength was measured and is shown in Fig. 11a. The onset of IPCE was around 475 nm and the observed IPCE reached 12% for 400 nm (comparable to the value reported by Zhang *et al.*^51^) and almost 40% for 300 nm. A Tauc plot for an indirect transition was calculated as (ln(1/(1 − IPCE)) × *hν*)^1/2^ and shown in Fig. 11b. The extrapolated value of the Tauc function yielded a band gap of 2.58 eV which is in agreement with previously reported values, *e.g.* 2.7 eV by Butler^17^ and Zhang *et al.*^51^ Tauc plots for WO_3_ films with the presence of Cl did not show any shift of the bandgap.

## 4. Conclusions

This work systematically investigated the impact of various W precursors and Cl doping (*via* NH_4_Cl) on the physical properties and photoelectrochemical (PEC) performance of spray pyrolytically deposited WO_3_ films. The choice of precursor had little influence on the photoelectrochemical response, as determined from long-term experiments, except in the case of films obtained from the WCl_6_ precursor, which exhibited comparatively lower photocurrent densities. All WO_3_ films showed good crystallinity. Films derived from WCl_6_ precursors were smoother, with MeOH-based solutions yielding lower photocurrents than EtOH-based solutions, except during the initial phase of amperometry.

The introduction of NH_4_Cl to the AMT precursor markedly enhanced the initial photocurrent density, likely due to reduced surface recombination resulting from oxidation of readily available chlorine species on top of the film as shown by topological EDS analysis. However, this enhancement was accompanied by a decrease in photocurrent during long-term polarization under illumination, presumably due to the depletion of chlorine as a hole scavenger in localized areas. In contrast, WO_3_ films prepared from pure AMT and PTA exhibited stable photocurrent generation over extended periods.

These findings highlight the complex interplay between precursor chemistry and photoelectrochemical behavior in sprayed WO_3_ thin films. Further optimization of deposition parameters and doping concentrations will be essential to fully exploit the potential of these materials for durable and efficient PEC applications. The superior long-term stability of WO_3_ films derived from AMT and PTA also indicates their suitability for applications requiring sustained performance.

## Conflicts of interest

There are no conflicts to declare.

## Supplementary Material

RA-016-D5RA07105D-s001

## Data Availability

The data presented in this study are available at https://doi.org/10.5281/zenodo.16897650. Data set for “WO_3_ electrodes by spray pyrolysis for photoelectrochemical applications: impact of W precursor and Cl incorporation” (Original data) (Zenodo). Supplementary information (SI) is available. See DOI: https://doi.org/10.1039/d5ra07105d.
