# Supplementary material for: WO3 electrodes by spray pyrolysis for photoelectrochemical applications: impact of W precursor and Cl incorporation
Source: RSC Adv. 2026 Jan 16;16(4):3554–63. doi: 10.1039/d5ra07105d (PMC12809387; doi:10.1039/d5ra07105d)
Supplement: RA-016-D5RA07105D-s001 [file RA-016-D5RA07105D-s001.pdf]

Supplementary Information (SI) for RSC Advances.
This journal is © The Royal Society of Chemistry 2026

Jk130126

Supplementary information

WO_3 electrodes by spray pyrolysis for photoelectrochemical applications: Impact of W precursor and Cl incorporation

Mohammed M. Gomaa [a], Hana Krýsová [b], Mohamed H. Sayed [a,c], Tomáš Imrich [b,d], Mostafa Boshta [a], Michael Neumann-Spallart d, Josef Krýsa [d,*]

[a] Solid State Physics Department, National Research Centre, 12622 Dokki, Giza, Egypt.
[b] J. Heyrovský Institute of Physical Chemistry, Czech Academy of Sciences, Dolejškova 2155/3, 182 23, Prague 8, Czech Republic.
[c] Molecular and Fluorescence Spectroscopy Lab., Central Laboratories Network, National Research Centre, 12622 Dokki, Giza, Egypt.
[d] Department of Inorganic Technology, University of Chemistry and Technology Prague, Technická 5, 166 28 Prague 6, Czech Republic.

Table S1. Comparison of photocurrents of WO_3 films prepared from AMT, PTA, WCl_6 in EtOH and WCl_6 in MeOH precursors at 1.4 V *vs.* Ag/AgCl under monochromatic illumination at 369 nm, 100 W/m^2.

Precursor solution	Layer thickness (µm)	Photocurrent density @ 1.4V *vs.* Ag/AgCl (mA/cm^2)
AMT	0.58	0.653
AMT	1.47	0.294
AMT + NH_4Cl (3 % Cl)	0.64	2.82
AMT + NH_4Cl (3 % Cl)	1.48	3.1
AMT + NH_4Cl (6 % Cl)	0.58	1.72
AMT + NH_4Cl (6 % Cl)	1.69	2
PTA	0.56	0.43
PTA	1.09	0.5
PTA	1.49	0.37
PTA	2.19	0.44
PTA	2.55	0.59
PTA	2.99	0.73
WCl_6 in MeOH	0.51	1.08
WCl_6 in MeOH	0.96	1.02
WCl_6 in MeOH	1.11	1.02
WCl_6 in EtOH	0.21	0.25
WCl_6 in EtOH	0.46	0.29
